# Efficacy of nasal high-flow therapy versus standard oxygen therapy in hypoxemic respiratory failure: a systematic review and meta-analysis of RCTS

**DOI:** 10.1097/MS9.0000000000004736

**Published:** 2026-07-03

**Authors:** Naveed Ahmed Khan, Furqan Ahmad Sethi, Khizar Yasin, Mustafa Zuhair Arshad, Ahmad Khan, Zarar Ahmad Khan Afridi, Umama Alam, Humam Shah, Rudaina Hanif, Aaima Zain, Warda Baber, Muhammad Abdullah Ali, Zaryab Bacha, Muhammad Paris Tamoor, Hassaan Abid, Saad Ali Shah, Kamil Ahmad Kamil

**Affiliations:** a Department of Medicine, Khyber Medical College, Peshawar, Pakistan; b Department of Medicine, Ayub Medical College Abbottabad; c Department of Medicine, Indiana University School of Medicine; d Department of Medicine, Bacha Khan Medical College, Mardan, Peshawar; e Internal Medicine Department, Nasrat Bashir Curative Hospital, Kandahar, Afghanistan

## Abstract

**Introduction:**

Respiratory failure is a serious condition caused by impaired gas exchange, leading to hypoxemia or hypercapnia. Nasal high-flow therapy (NHFT) has emerged as an alternative to standard oxygen therapy (SOT). This meta-analysis evaluates the efficacy and safety of NHFT versus SOT by incorporating newly published trials to guide current clinical practice.

**Methods:**

This systematic review and meta-analysis followed PRISMA and Cochrane guidelines. Randomized controlled trials (RCTs) comparing NHFT with SOT in acute respiratory failure were included. Primary outcomes were mortality and hospital stay. Data were pooled using a random-effects model in RevMan 5.4, and the certainty of evidence was assessed using GRADE.

**Results:**

Sixteen RCTs (*n* = 5805) were included. NHFT showed no significant difference in 28-day mortality [risk ratio (RR) = 1.00; 95% confidence interval (CI): 0.85–1.17] or 90-day mortality (RR = 0.87; 95% CI: 0.58–1.29), length of hospital stay, length of ICU stay, or need for intubation. Oxygen saturation improved with NHFT after sensitivity analysis. Certainty of evidence ranged from moderate to very low using GRADE assessment, with heterogeneity addressed by removing outlier studies.

**Conclusion:**

NHFT shows comparable safety and efficacy to SOT in acute hypoxemic respiratory failure, with no significant differences in mortality, length of hospital stay, or intubation rates. Oxygen saturation improved with NHFT, reflecting its physiological advantages. Variability in study design limits generalizability. These findings support the individualized use of NHFT. Future research should explore patient-specific benefits, standardize protocols, and assess long-term outcomes.

## Introduction

The respiratory system enables gas exchange between the body and the external environment, a process essential for aerobic metabolism. It delivers oxygen and eliminates carbon dioxide; failure to perform either function leads to respiratory failure. Type 1 respiratory failure is characterized by inadequate oxygenation (hypoxemia), while Type 2 involves insufficient carbon dioxide elimination (hypercapnia). Respiratory failure can also be classified by its duration as acute, chronic, or acute-on-chronic^[^1^]^. In 2017, the incidence of respiratory failure in the United States was reported as 1275 cases per 100 000 adults^[^2^]^. The global incidence of acute respiratory failure caused by acute respiratory distress syndrome (ARDS) varies widely, ranging from 10 to 80 cases per 100 000 people per year, depending on the region^[^3^]^. Approximately 79% of hospitalized COVID-19 patients developed respiratory failure^[^4^]^. Additionally, acute exacerbation of chronic obstructive pulmonary disease (COPD) is the third most common cause of hospitalization due to acute respiratory failure^[^5^]^.

HIGHLIGHTS

- Compared NHFT with SOT in acute hypoxemic respiratory failure across 16 RCTs.
- NHFT showed no difference in mortality, hospital stay, or intubation rates.
- Oxygen saturation significantly improved with NHFT versus SOT.
- Evidence certainty ranged from moderate to very low using the GRADE assessment.
- Supports individualized NHFT use as a safe alternative to conventional therapy.

Oxygen therapy is the primary treatment for patients with hypoxemia. Delivery devices are generally categorized as low-flow or high-flow systems. Low-flow devices – such as nasal catheters, cannulas, and simple face masks – provide a limited oxygen flow (typically 1–15 L/min) that mixes with the patient’s inspiratory airflow^[^6^]^. However, standard oxygen therapy (SOT) is often linked to variable inspired oxygen fractions (FiO₂), dryness of the nasal and oral mucosa, nasal bleeding, and poor tolerance. Additionally, studies have shown that 7–15% of COPD patients with acute exacerbations receiving SOT eventually require invasive mechanical ventilation, a marker of significantly increased mortality risk^[^7,8^]^. However, evidence regarding the overall impact of noninvasive ventilation on preventing intubation and improving clinical outcomes remains inconsistent^[^9,10^]^.

Nasal high-flow therapy (NHFT) is a relatively recent respiratory support modality that has gained widespread use in adult patients with acute respiratory failure over the past decade^[^11^]^. NHFT delivers heated and fully humidified gas at high flow rates, which preserves mucociliary function and limits airway drying and resistance. The elevated flow washes out CO₂ from the upper airway, reducing anatomical dead space and thereby decreasing the work of breathing^[^12^]^. Furthermore, the high flow generates a low level of positive end-expiratory pressure (PEEP) that increases end-expiratory lung volume, promotes alveolar recruitment, and improves ventilation homogeneity^[^13^]^. NHFT enhances oxygenation and patient comfort by delivering warm, humidified gas at high flow rates, which reduces airway dryness and improves tolerance. The high flow helps flush out anatomical dead space, leading to more efficient ventilation. Additionally, the mild PEEP effect helps maintain alveolar recruitment and improves gas exchange. In patients with acute respiratory failure from diverse causes, NHFT has been shown to provide greater comfort and improved oxygenation compared to standard oxygen delivered via face mask^[^14^]^. Conversely, a randomized clinical trial reported a reduced risk of intubation with NHFT compared to SOT but found no significant difference in mortality^[^15^]^. A recent clinical trial, however, reported no significant difference in intubation rates between NHFT and SOT^[^16^]^. These discrepancies underscore the conflicting nature of the current evidence.

Given the conflicting findings across studies, comparing NHFT and SOT in patients with acute respiratory failure remains uncertain. While some studies suggest improved oxygenation, greater patient comfort, and reduced need for intubation with NHFT, others report no significant differences in major clinical outcomes, including mortality. The most comprehensive prior meta-analysis by Rochwerg *et al*^[^17^]^ evaluated high-flow nasal cannula (HFNC) compared with conventional oxygen therapy in acute hypoxemic respiratory failure and demonstrated a reduced risk of intubation but no mortality benefit. However, most of the included trials were small, unblinded, and underpowered, limiting the strength and precision of the pooled estimates. Although several subgroup analyses were planned – including by severity of hypoxemia and patient setting – only limited analyses could be performed because of insufficient data. To address these limitations, the present meta-analysis incorporates recently published randomized controlled trials (RCTs) with larger sample sizes, performs refined subgroup analyses based on the severity of hypoxemia, and applies the GRADE framework to assess the certainty of evidence. This updated meta-analysis aims to provide a more statistically robust and clinically meaningful synthesis to clarify the role of HFNC in the management of acute respiratory failure, particularly its impact on key clinical outcomes such as mortality, hospital length of stay, intubation rates, oxygenation, need for invasive ventilation, heart rate, and incidence of cardiac arrest – findings that are essential for guiding evidence-based clinical practice. This manuscript adheres to the TITAN guidelines for the transparent reporting of artificial intelligence use in scholarly research^[^18^]^.

## Methods

### Study design

This study was structured as a systematic review and meta-analysis, following the methodologies outlined in the Cochrane Handbook for Systematic Reviews of Interventions^[^19^]^ and adhering to the reporting standards specified in the Preferred Reporting Items for Systematic Reviews and Meta-Analyses (PRISMA)^[^20^]^. The protocol for this review was formally registered in the International Prospective Register of Systematic Reviews (CRD420251103622). The work has been reported in line with AMSTAR (Assessing the Methodological Quality of Systematic Reviews) guidelines.

### Literature search and strategy

An extensive database search was performed using PubMed, Embase, and Scopus, collecting all articles from their inception up to May 2025. The search was limited to studies published in the English language. Additionally, a detailed examination of the reference lists of included studies and relevant systematic reviews was carried out to identify applicable studies. The search strategy integrated Medical Subject Headings (MeSH) and keywords associated with “Nasal High-Flow Therapy” and “Standard Oxygen Therapy.” A detailed search strategy is provided as Supplemental Digital Content Table 2, available at: http://links.lww.com/MS9/B247.

### Eligibility criteria

The included studies met the following characteristics^[^1^]^: study design: RCTs to ensure high-quality evidence^[^2^]^; population: patients of any age requiring respiratory support due to acute hypoxemic, hypercapnic, or mixed respiratory failure, with conditions such as acute respiratory failure or post-extubation care^[^3^]^; intervention: NHFT for respiratory support^[^4^]^; comparator: SOT or conventional care/control group^[^5^]^; language: studies published in English.

In this meta-analysis, *SOT* was defined as the use of conventional low-flow oxygen delivery devices, such as a nasal cannula, simple face mask, or Venturi mask, as reported by the individual studies. These devices typically deliver oxygen at flow rates between 1 and 15 L/min, resulting in variable inspired oxygen fractions (FiO₂), depending on the patient’s breathing pattern.

Non-randomized studies, case series, review articles, and letters to the editor were excluded from our analysis.

### Study selection and data extraction

Studies identified through our extensive search strategy were imported into Rayyan software, where duplicate records were eliminated. Two independent reviewers then carefully assessed the remaining studies by screening titles and abstracts against predefined inclusion criteria, with any discrepancies resolved through discussion with a senior author. The extracted data included baseline characteristics such as study ID, country, type of study, sample size, and proportion of male participants. Additionally, details were gathered on the type of intervention and control group. A full-text review of the selected articles was performed to confirm compliance with our inclusion criteria.

Data from the included studies were extracted by two reviewers independently into a pre-designed Excel spreadsheet. Any disagreements were addressed through consultation with a senior author. Dichotomous outcomes were reported as event counts and total participants, while continuous outcomes were presented as means with corresponding standard deviations (SD).

### Outcomes

The primary outcomes were length of hospital stay, 28-day mortality, 90-day mortality, and in-hospital mortality. The secondary outcomes were length of ICU stay (days), cardiac arrest, need for intubation (procedural event), need for invasive ventilation (post-intubation), need for non-invasive ventilation (NIV), dyspnea (measured by Visual Analog Scale – VAS), patient comfort, heart rate, and oxygen saturation (SpO₂). For detailed definitions of outcomes, refer to Supplemental Digital Content Table 3, available at: http://links.lww.com/MS9/B247

### Risk of bias assessment

Two reviewers independently evaluated the risk of bias in the included studies, with any discrepancies resolved through discussion with a third author. The Cochrane Risk of Bias version 2 (ROB-2) tool was employed for assessing RCTs. This tool examines bias across five key domains^[^1^]^: bias arising from the randomization process^[^2^]^; bias due to deviations from the intended interventions^[^3^]^; bias resulting from missing outcome data^[^4^]^; bias related to the measurement of outcomes; and^[^5^]^ bias due to the selection of reported results. All included RCTs were assessed using this framework and categorized as having low risk, high risk, or some concerns.

### Statistical analysis

Statistical analysis was conducted using Review Manager version 5.4. A random-effects model was applied to perform the meta-analyses in order to account for variability among the included studies. Pooled effect estimates were reported as risk ratios (RRs) with corresponding 95% confidence intervals (CIs) for dichotomous outcomes, while mean differences were used for continuous outcomes.

Heterogeneity among studies was assessed using the *I*^2^ statistic, with values of 25, 50, and 75% indicating low, moderate, and high heterogeneity, respectively. In cases where considerable heterogeneity was detected (*I*^2^ > 50%) for primary outcomes, sensitivity analyses were conducted to explore potential sources. Outlier studies were identified using leave-one-out sensitivity analysis and defined as those contributing >50% to heterogeneity (*I*^2^) or deviating by more than two SDs from the pooled effect estimate. When excluded, heterogeneity was reassessed, and changes in pooled estimates were reported.

Publication bias was considered but not formally assessed using funnel plots or Egger’s test, as most pooled outcomes included fewer than 10 studies, making such analyses unreliable.

A subgroup analysis was conducted by categorizing patients into mild and severe hypoxemic respiratory failure according to the PaO_2_/FiO_2_ ratios and clinical parameters defined in the included studies.

### Grade assessment

The certainty of evidence in this meta-analysis was assessed using the Grading of Recommendations, Assessment, Development, and Evaluation (GRADE) approach, specifically through the GRADEpro Guideline Development Tool^[^21^]^. Two independent reviewers evaluated the evidence and categorized it as high, moderate, low, or very low certainty^[^22^]^. Any disagreements were resolved through discussion and mutual consensus.

## Results

### Search results

**Figure 1.**
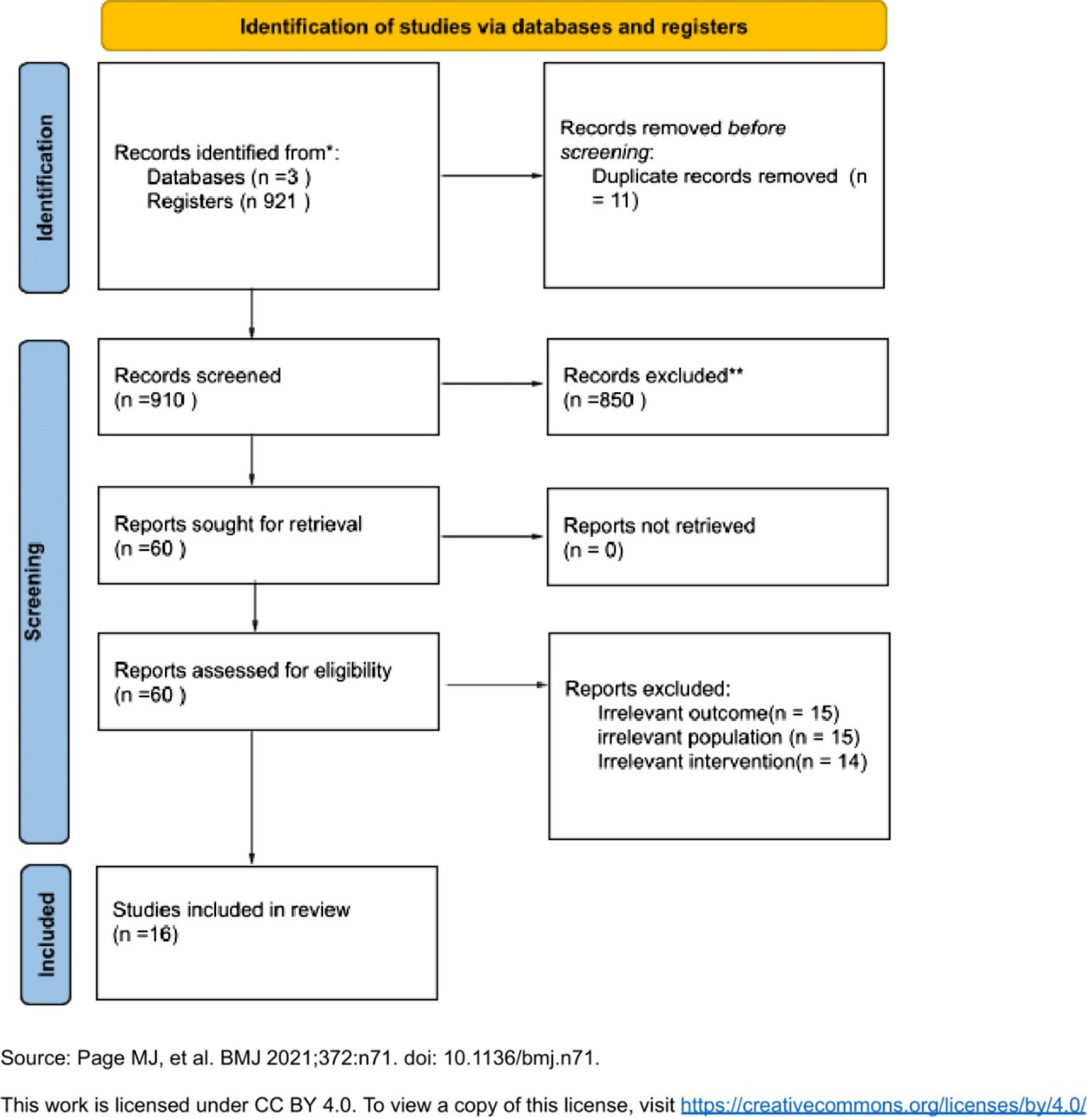
PRISMA flow diagram illustrating the study selection process for the meta-analysis. Out of 924 initially identified records, 16 randomized controlled trials were included after screening and full-text review.

A total of 921 records were identified through a comprehensive database and registry search. After removing 11 duplicates, 910 records were screened by title and abstract. Of these, 850 were excluded, and 60 full-text articles were assessed for eligibility. The remaining 44 articles were excluded due to irrelevant outcomes (*n* = 15) or non-eligible population/intervention (*n* = 29). Ultimately, 16^[^23–38^]^ studies involving 5805 participants met the inclusion criteria and were included in the final analysis. The study selection process is detailed in the PRISMA flow diagram (Fig. 1).

### Study characteristics

A total of 5805 patients were included, with 2911 in the NHFT group and 2894 in the SOT group. The studies, published between 2011 and 2025, included two each from Australia, New Zealand, and Thailand; others were conducted in France, Italy, Germany, China, Turkey, Belgium (with France), and multinational combinations involving Australia, New Zealand, and Switzerland.

Primary outcomes were length of hospital stay, 28-day mortality, and 90-day mortality. Secondary outcomes included ICU stay, cardiac arrest, intubation, invasive and NIV, dyspnea (VAS), patient comfort, heart rate, and oxygen saturation (SpO₂).

Across the included studies, the mean or median age of participants ranged from approximately 50–70 years, reflecting a predominantly adult population. The severity of hypoxemia at baseline varied, with most studies enrolling patients with a PaO₂/FiO₂ ratio below 300 mmHg, consistent with moderate hypoxemic respiratory failure. Several trials also included patients with hypercapnic or mixed respiratory failure, particularly those with acute exacerbations of COPD. Common comorbidities reported across studies included hypertension, diabetes mellitus, and chronic lung disease. These variations reflect the broad clinical spectrum represented in the pooled population and enhance the external validity of our findings. Baseline characteristics are detailed in Tables 1 and 2

**Table 1:** Study characteristics.

Study ID	Country	Type of study	Intervention	Control	Sample size(*n*)		Male (%)	
					Nasal high flow	Standard oxygen therapy	Nasal high flow	Standard oxygen therapy
Azoulay *et al*^[^27^]^	France	RCT	Nasal high flow	Standard oxygen therapy	388	388	69.6	63.6
Bell *et al*^[^28^]^	Australia	RCT	Nasal high flow	Standard oxygen therapy	48	52	46.2	41.7
Crimi 2022	Italy	RCT	Nasal high flow	Standard oxygen therapy	181	181	65.7	61.9
Franklin *et al*^[^29^]^	Australia, New Zealand	RCT	Nasal high flow	Standard oxygen therapy	753	764	49.8	53.7
Frat *et al*^[^31^]^	France	RCT	Nasal high flow	Standard oxygen therapy	357	354	70	70
Frat *et al*^[^30^]^	Belgium, France	RCT	Nasal high flow	Standard oxygen therapy	106	94	71	67
George *et al*^[^33^]^	Australia, New Zealand, Switzerland	RCT	Nasal high flow	Standard oxygen therapy	480	481	61.2	61.5
Jones 2015	New Zealand	RCT	Nasal high flow	Standard oxygen therapy	165	138	44.2	51.5
Lemiale *et al*^[^24^]^	France	RCT	Nasal high flow	Standard oxygen therapy	52	48	73.1	66.7
Makdee *et al*^[^35^]^	Thailand	RCT	Nasal high flow	Standard oxygen therapy	63	65	NR	NR
Mendil 2021	Turkey	RCT	Nasal high flow	Standard oxygen therapy	51	49	65	67
Parke *et al*^[^34^]^	New Zealand	RCT	Nasal high flow	Standard oxygen therapy	56	56	23	21
Rittayamai *et al*^[^36^]^	Thailand	RCT	Nasal high flow	Standard oxygen therapy	20	20	NR	NR
Ruangsomboom 2020	Thailand	RCT	Nasal high flow	Standard oxygen therapy	19	18	NR	NR
Schwabbauer *et al*^[^23^]^	Germany	RCT	Nasal high flow	Standard oxygen therapy	14	14	NR	NR
Xia *et al*^[^25^]^	China	RCT	Nasal high flow	Standard oxygen therapy	158	172	88.6	79.7

**Table 2:** Patient characteristics.

	Age, mean (SD)		BMI, mean (SD)		Hypertension, *n* (%)		Diabetes, *n* (%)		Asthma, *n* (%)		Chronic kidney disease, *n* (%)		Cardiovascular disease, *n* (%)	
Study ID	Nasal high flow	Standard oxygen therapy	Nasal high flow	Standard oxygen therapy	Nasal High flow	Standard oxygen therapy	Nasal high flow	Standard oxygen therapy	Nasal high flow	Standard oxygen therapy	Nasal high flow	Standard oxygen therapy	Nasal high flow	Standard oxygen therapy
Azoulay *et al*^[^27^]^	63	11.16	NR	NR	NR	NR	NR	NR	NR	NR	73(18.8)	69(20.4)	NR	NR
Bell *et al*^[^28^]^	74.5(14)	72.9(15.1)	NR	NR	NR	NR	NR	NR	NR	NR	NR	NR	NR	NR
Crimi 2022	59.01(14.88)	58.92(14.77)	28.55(4.33)	27.99(4.45)	NR	NR	18(9.8)	26(14.2)	NR	NR	6(3.3)	6(3.3)	11(6.1)	10(5.5)
Franklin *et al*^[^29^]^	2.10(1.19)	2.07(1.11)	NR	NR	NR	NR	NR	NR	236(31.3)	255(33.4)	NR	NR	11(1.5)	15(2.0)
Frat *et al*^[^31^]^	61(12)	61(12)	29(5)	29(6)	NR	NR	NR	NR	NR	NR	NR	NR	31(9)	32(9)
Frat *et al*^[^30^]^	61(16)	59(17)	25(5)	26(5)	NR	NR	NR	NR	NR	NR	NR	NR	NR	NR
George *et al*^[^33^]^	1.41(2.65)	1.36(2.54)	NR	NR	NR	NR	NR	NR	NR	NR	NR	NR	NR	NR
Jones 2015	74.6(15.6)	72.2(16.8)	NR	NR	NR	NR	NR	NR	7(4.2)	13(9.4)	NR	NR	NR	NR
Lemiale *et al*^[^24^]^	57.43(20.59)	63.25(14.33)	NR	NR	NR	NR	NR	NR	NR	NR	2(3.8)	3(6.2)	NR	NR
Makdee *et al*^[^35^]^	70(15)	71.2(14.2)	NR	NR	51(81)	41(63.1)	29(46)	29(44.6)	NR	NR	17(27)	17(26.2)	NR	NR
Mendil 2021	58.5(13.532)	58(13.532)	NR	NR	NR	NR	NR	NR	NR	NR	NR	NR	NR	NR
Parke *et al*^[^34^]^	64(13.098)	64(9.768)	NR	NR	NR	NR	NR	NR	NR	NR	NR	NR	NR	NR
Rittayamai *et al*^[^36^]^	65.6(14.4)	63.5(15.7)	NR	NR	10(50)	10(50)	5(25)	9(45)	2(10)	5(25)	NR	NR	7(35)	3(15)
Ruangsomboom 2020	63.7(16.9)	63.2(21.8)	NR	NR	10(52.6)	5(27.8)	5(26.3)	2(11.1)	NR	NR	3(15.8)	0(0)	2(10.5)	1(5.6)
Schwabbauer *et al*^[^23^]^	NR	NR	NR	NR	NR	NR	NR	NR	NR	NR	NR	NR	NR	NR
Xia *et al*^[^25^]^	70(7.48)	69(8.22)	21(3.44)	21(3.44)	54(34.2)	65(37.8)	16(10.1)	13(7.6)	11(7)	8(4.7)	NR	NR	7(4.4)	12(7.0)

NR, Not reported.

.

### Quality assessment

Risk of bias was assessed using the Cochrane RoB 2 tool. Domains D1 and D3 showed low risk across all studies. D4 raised concerns in all studies (Mendil 2021, Crimi *et al*^[^37^]^, George *et al*^[^33^]^, Frat *et al*^[^31^]^, Franklin *et al*^[^29^]^, Ruangsomboon *et al*^[^26^]^, Xia *et al*^[^25^]^, Bell *et al*^[^28^]^, Parke *et al*^[^34^]^, Makdee *et al*^[^35^]^, Rittayamai *et al*^[^36^]^) due to potential measurement issues. D2 showed concerns in Schwabbauer *et al*^[^23^]^, Azoulay *et al*^[^27^]^, Makdee *et al*^[^35^]^, Frat *et al*^[^30^]^, Mendil 2021, and Crimi *et al*^[^27^]^ due to possible protocol deviations. D5 raised concerns in Mendil 2021, Schwabbauer *et al*^[^23^]^, and Lemiale *et al*^[^24^]^ due to unclear outcome reporting. Full details are provided in the Supplemental Digital Content Figure 1, available at: http://links.lww.com/MS9/B246.

### Grade assessment

Using the GRADEpro Guideline Development Tool, the GRADE framework was employed to evaluate the certainty of evidence across the analyzed outcomes. The overall quality of evidence ranged from moderate to very low. Moderate-certainty evidence supported the outcomes of length of ICU stay, in-house mortality, 28-day mortality, and need for NIV. In contrast, the certainty of evidence for length of hospital stay, invasive ventilation, comfort scale score, cardiac arrest, and oxygen saturation (SpO₂) was rated as low. Very low certainty was assigned to outcomes including intubation, heart rate, dyspnea (VAS score), and 90-day mortality. Downgrading decisions were primarily based on methodological limitations, such as serious risk of bias due to open-label study designs and inadequate blinding, inconsistency in study results, and imprecision resulting from low event rates and wide confidence intervals. The assessment of publication bias was limited due to the small number of trials included for several outcomes, although no serious concerns were detected in this domain. For details, refer to Supplemental Digital Content Table 1, available at: http://links.lww.com/MS9/B247

### Meta-analysis of primary outcomes

#### Length of hospital stay

**Figure 2.**
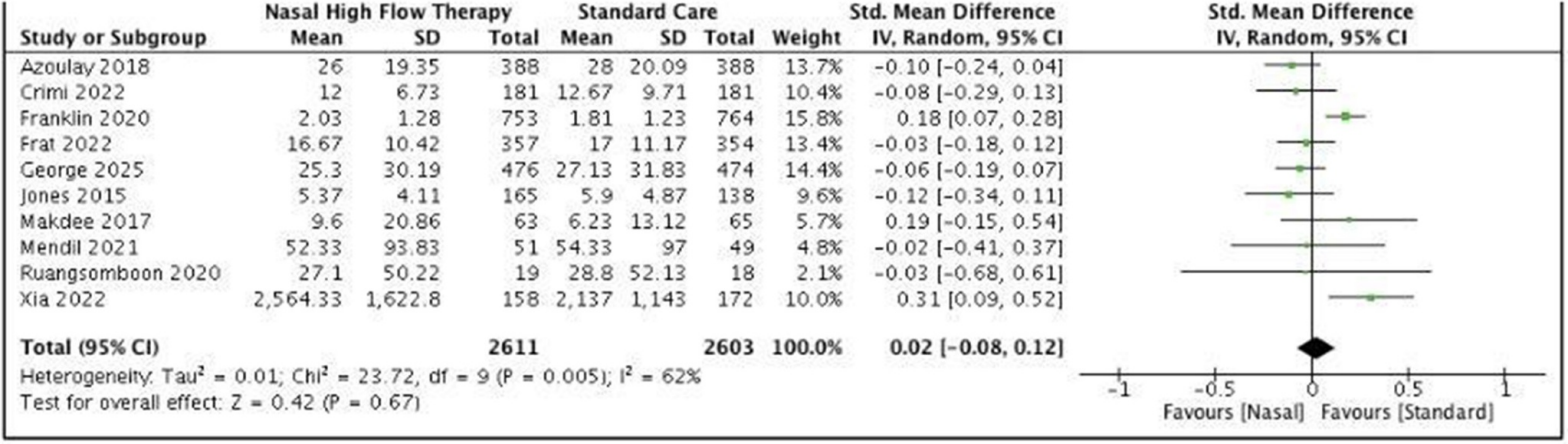
Forest plot showing the efficacy of nasal high-flow therapy versus standard oxygen therapy in hypoxemic respiratory failure in terms of length of hospital stay (SMD: 0.02; 95% CI: −0.08 to 0.12; *P* = 0.67; *I*^2^ = 62%).

There was no significant difference in hospital stay between the NHFT and SOT groups [standardized mean difference (SMD): 0.02; 95% CI: −0.08 to 0.12; *P* = 0.67; *I*^2^ = 62%)] (Fig. 2). Sensitivity analysis excluding Franklin *et al*^[^29^]^ confirmed the result (SMD: −0.01; 95% CI: −0.10 to 0.07; *P* = 0.76), with reduced heterogeneity (*I*^2^ = 37%) (Supplemental Digital Content Figure 2, available at: http://links.lww.com/MS9/B246)

#### Subgroup analysis

A subgroup analysis was performed to assess whether the severity of hypoxemia influenced the length of hospital stay among patients receiving NHFT or SOT.

**Figure 3.**
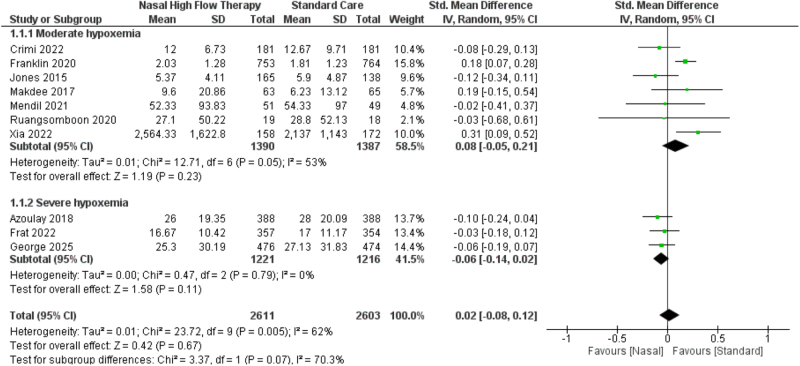
Subgroup analysis of length of hospital stay.

In the moderate hypoxemia subgroup, the pooled SMD was 0.08 (95% CI: −0.05 to 0.21; *I*^2^ = 53%), while in the severe hypoxemia subgroup, the SMD was −0.06 (95% CI: −0.14 to 0.02; *I*^2^ = 0%), both showing no significant differences between treatment groups. The overall pooled estimate indicated no difference in hospital stay duration [SMD: 0.02 (95% CI: −0.08 to 0.12; *I*^2^ = 62%)] (Fig. 3).

Following sensitivity analysis that excluded studies contributing to heterogeneity, the results remained consistent (SMD: −0.01 [95% CI: −0.10 to 0.07; *I*^2^ = 37%]), confirming the robustness of the findings (Supplemental Digital Content Figure 6, available at: http://links.lww.com/MS9/B246). No significant subgroup interaction was detected (*P* = 0.07; *I*^2^ = 70.3%), suggesting that the effect of NHFT on hospital length of stay was consistent across varying severities of hypoxemia.

#### 28-day mortality

**Figure 4.**
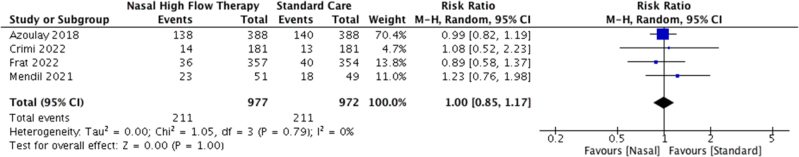
Forest plot showing the efficacy of nasal high-flow therapy versus standard oxygen therapy in hypoxemic respiratory failure in terms of 28-day mortality (RR: 1.00; 95% CI: 0.85–1.17; P = 1.00).

The 28-day mortality rates did not differ significantly between the NHFT and SOT groups (RR: 1.00; 95% CI: 0.85–1.17; P = 1.00). Heterogeneity between the studies was low (*I*^2^ = 0%) (Fig. 4).

#### Subgroup analysis

To explore whether the severity of hypoxemia influenced treatment outcomes, a subgroup analysis of 28-day mortality was carried out, classifying studies into severe and moderate hypoxemia groups. Across both subgroups, no significant difference was observed.

**Figure 5.**
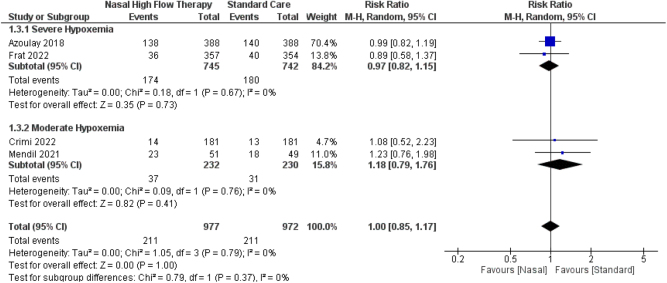
Subgroup analysis of 28-day mortality.

In patients with severe hypoxemia, the pooled RR was 0.97 (95% CI: 0.82–1.15; *I*^2^ = 0%), while those with moderate hypoxemia demonstrated a RR of 1.18 (95% CI: 0.79–1.76; *I*^2^ = 0%). No significant heterogeneity was detected within or between subgroups (*P* = 0.37; *I*^2^ = 0%). These findings suggest that the severity of hypoxemia did not significantly modify the effect of NHFT on 28-day mortality, and outcomes were consistent across varying degrees of baseline oxygen impairment (Fig. 5).

#### 90-day mortality

**Figure 6.**
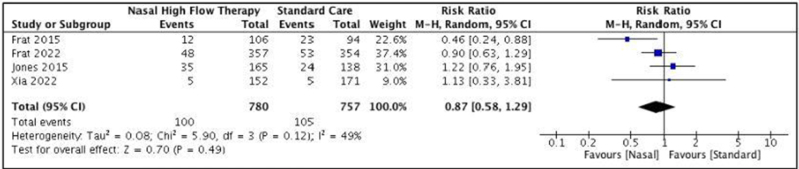
Forest plot showing the efficacy of nasal high-flow therapy versus standard oxygen therapy in hypoxemic respiratory failure in terms of 90-day mortality (RR: 0.87; 95% CI: 0.58–1.29; *P* = 0.49).

The 90-day mortality rates did not differ significantly between the NHFT and SOT groups (RR: 0.87; 95% CI: 0.58–1.29; *P* = 0.49). Heterogeneity between the studies was moderate (*I*^2^ = 49%) (Fig. 6).

#### Subgroup analysis

A subgroup analysis was performed to assess the severity of hypoxemia in influencing 90-day mortality outcomes. Among patients with moderate hypoxemia, the pooled RR for mortality was 1.21 (95% CI: 0.78–1.87; *I*^2^ = 0%), indicating no significant difference. In the severe hypoxemia subgroup, the pooled RR was 0.68 (95% CI: 0.36–1.29; *I*^2^ = 68%), also showing no statistically significant variability. The overall pooled estimate across all studies demonstrated a RR of 0.87 (95% CI: 0.58–1.29; *I*^2^ = 49%).

No significant interaction was observed between subgroups (*P* = 0.15; *I*^2^ = 52.2%).

**Figure 7.**
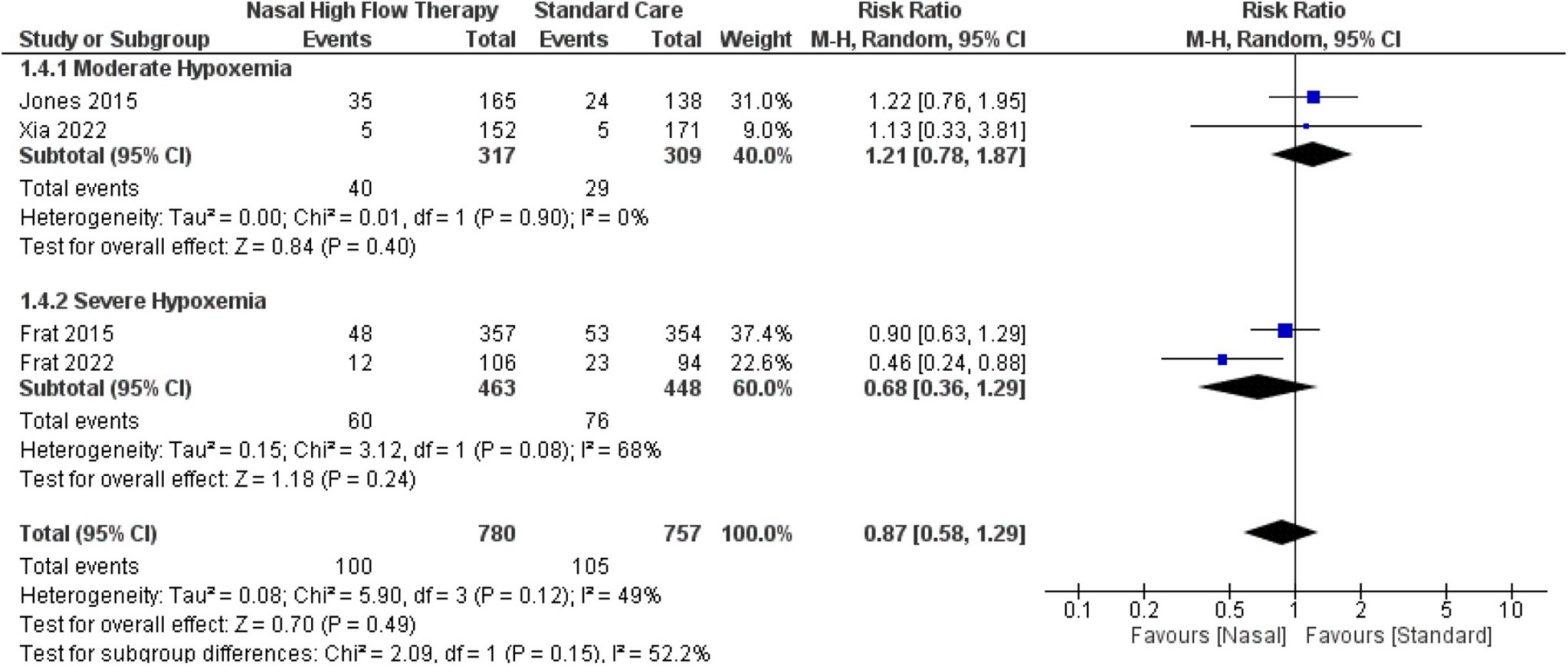
Subgroup analysis of 90-day mortality.

These findings suggest that the effect of NHFT on 90-day mortality was consistent across different severities of hypoxemia (Fig. 7).

#### In-hospital mortality

**Figure 8.**
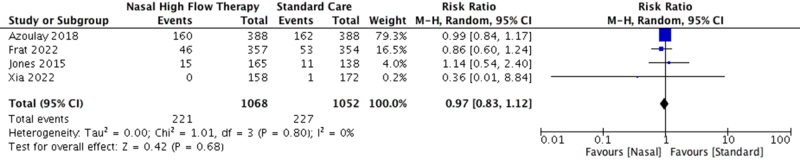
Forest plot showing the efficacy of nasal high-flow therapy versus standard oxygen therapy in hypoxemic respiratory failure in terms of in-hospital mortality (RR: 0.97; 95% CI: 0.83–1.12; *P* = 0.68).

The in-hospital mortality rates did not differ significantly between the NHFT and SOT groups (RR: 0.97; 95% CI: 0.83–1.12; *P* = 0.68). Heterogeneity between the studies was low (*I*^2^ = 0%) (Fig. 8).

#### Subgroup analysis

A subgroup analysis was performed to evaluate whether the severity of hypoxemia influenced in-hospital mortality. In patients with moderate hypoxemia, the pooled RR was 1.08 (95% CI: 0.52–2.22; *I*^2^ = 0%), indicating no significant difference between the two groups.

Similarly, in the severe hypoxemia subgroup, the pooled RR was 0.96 (95% CI: 0.83–1.12; *I*^2^ = 0%), also showing no statistically significant difference.

**Figure 9.**
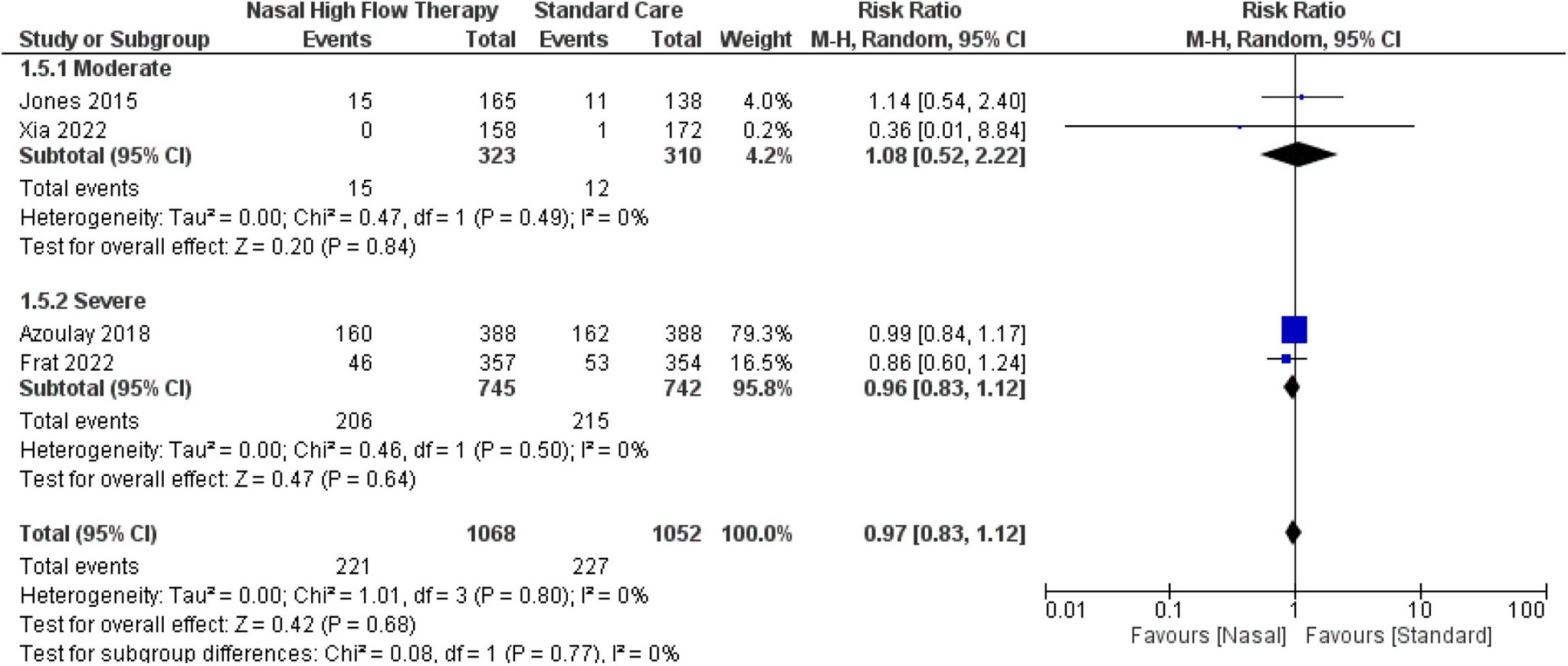
Subgroup analysis of in hospital mortality.

No significant heterogeneity or interaction was observed between subgroups (*P* = 0.77; *I*^2^ = 0%) (Fig. 9).

### Meta-analysis of secondary outcomes

#### Length of ICU stay days

**Figure 10.**
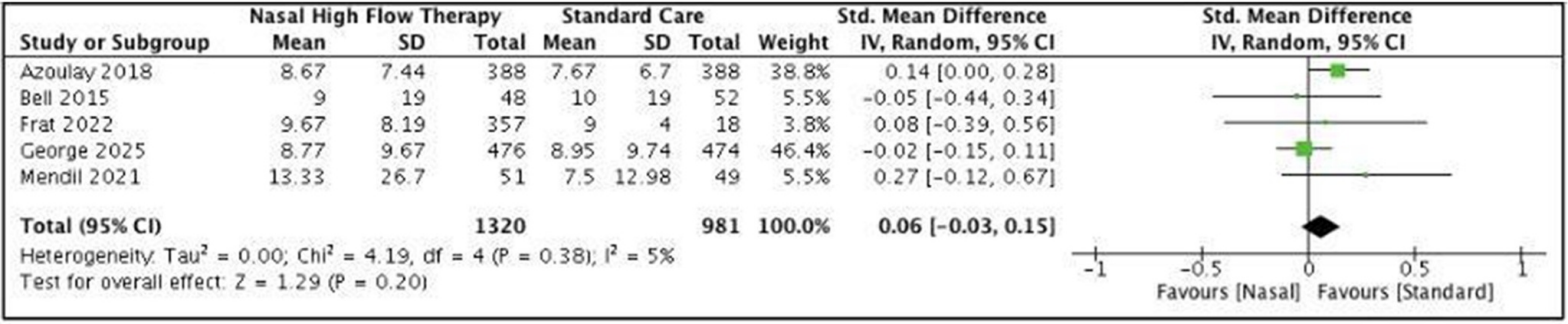
Forest plot showing the efficacy of nasal high-flow therapy versus standard oxygen therapy in hypoxemic respiratory failure in terms of length of ICU stay days (SMD: 0.06; 95% CI: −0.03 to 0.15; *P* = 0.20).

The length of ICU stay did not differ significantly between the NHFT and SOT groups (SMD: 0.06; 95% CI: −0.03 to 0.15; *P* = 0.20). Heterogeneity between the studies was low (*I*^2^ = 5%) (Fig. 10).

#### Subgroup analysis

**Figure 11.**
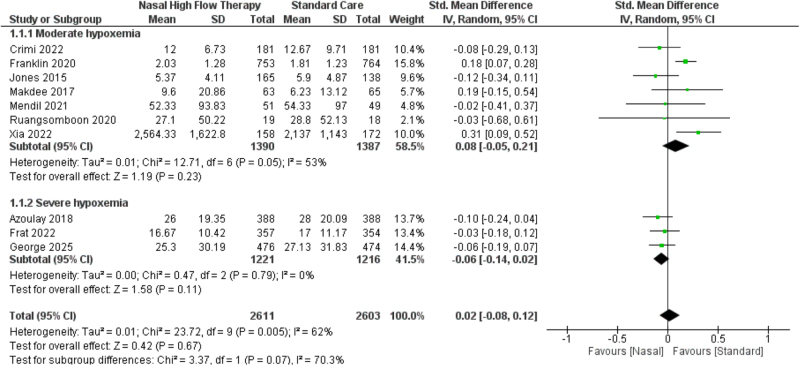
Subgroup analysis of length of ICU stay (days).

A subgroup analysis was performed to evaluate whether the severity of hypoxemia influenced the length of ICU stay. In patients with moderate hypoxemia, the pooled standardized mean difference (SMD) was 0.11 (95% CI: –0.21 to 0.43; *I*^2^ = 24%), while in the severe hypoxemia subgroup, the pooled SMD was 0.06 (95% CI: –0.06 to 0.18; *I*^2^ = 27%). No significant subgroup interaction was observed (*P* = 0.76; *I*^2^ = 0%), indicating that the effect of NHFT on ICU stay duration was consistent across varying severities of hypoxemia (Fig. 11).

#### Cardiac arrest

**Figure 12.**
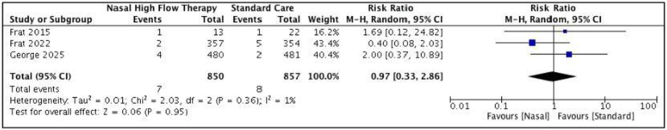
Forest plot showing the efficacy of nasal high-flow therapy versus standard oxygen therapy in hypoxemic respiratory failure in terms of cardiac arrest (RR: 0.97; 95% CI: 0.33–2.86; *P* = 0.95).

The risk of cardiac arrest did not differ significantly between the NHFT and SOT groups (RR: 0.97; 95% CI: 0.33–2.86; *P* = 0.95). Heterogeneity between the studies was low (*I*^2^ = 1%) (Fig. 12).

#### Need for intubation

**Figure 13.**
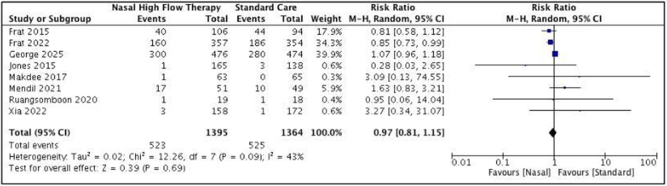
Forest plot showing the efficacy of nasal high-flow therapy versus standard oxygen therapy in hypoxemic respiratory failure risk of intubation (RR: 0.97; 95% CI: 0.81–1.15; *P* = 0.69.

The risk of intubation did not differ significantly between the NHFT and SOT groups (RR: 0.97; 95% CI: 0.81–1.15; *P* = 0.69). Heterogeneity between the studies was moderate (*I*^2^ = 43%) (Fig. 13).

#### Subgroup analysis

A subgroup analysis was conducted. In patients with moderate hypoxemia, the pooled RR was 1.51 (95% CI: 0.83–2.74; *I*^2^ = 0%), showing no significant difference between the two groups. For patients with severe hypoxemia, the pooled RR was 0.93 (95% CI: 0.77–1.12; *I*^2^ = 73%), also indicating no significant effect of NHFT on intubation risk.

**Figure 14.**
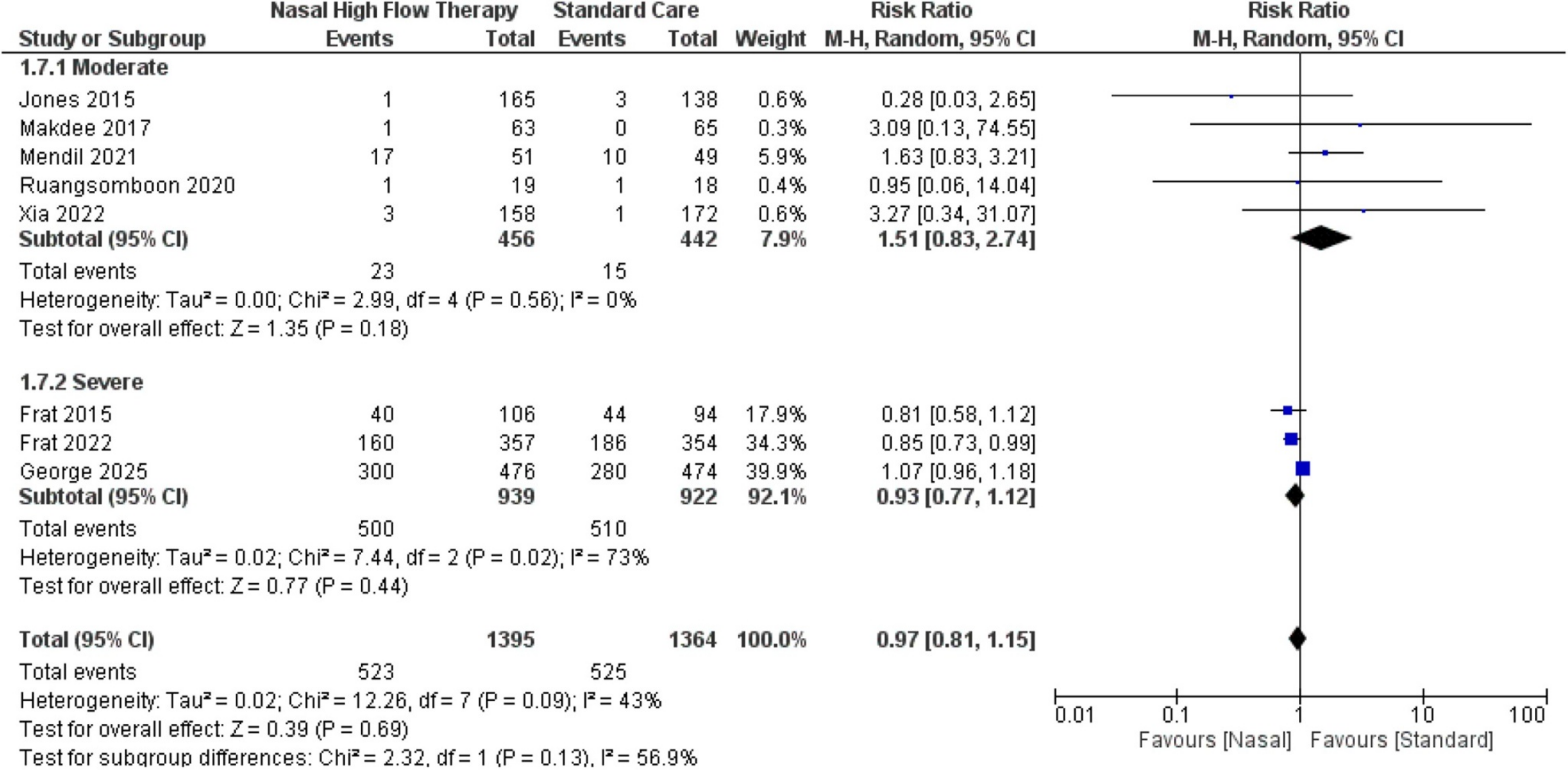
Subgroup analysis of need for intubation.

There was no significant subgroup difference based on hypoxemia severity (*P* = 0.13; *I*^2^ = 56.9%) (Fig. 14).

#### Need for invasive ventilation

**Figure 15.**
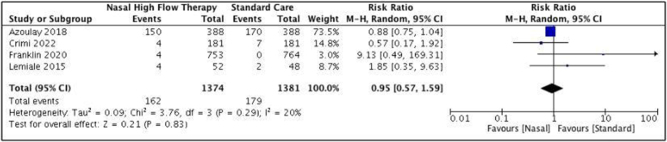
Forest plot showing the efficacy of nasal high-flow therapy versus standard oxygen therapy in hypoxemic respiratory failure in terms of need for invasive ventilation (RR: 0.95; 95% CI: 0.57–1.59; *P* = 0.83).

The need for invasive ventilation did not differ significantly between the NHFT and SOT groups (RR: 0.95; 95% CI: 0.57–1.59; *P* = 0.83). Heterogeneity between the studies was low (*I*^2^ = 20%) (Fig. 15).

#### Subgroup analysis

A subgroup analysis was conducted to evaluate whether the severity of hypoxemia influenced the need for invasive mechanical ventilation.

**Figure 16.**
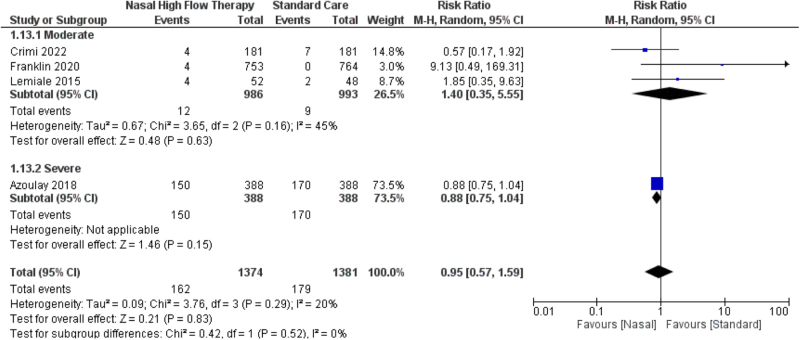
Subgroup analysis of need for invasive ventilation.

Among patients with moderate hypoxemia, the pooled RR was 1.40 (95% CI: 0.35–5.55; *I*^2^ = 45%), indicating no significant difference. Similarly, in the severe hypoxemia subgroup, the pooled RR was 0.88 (95% CI: 0.75–1.04), also showing no statistically significant difference. No significant subgroup interaction was detected (*P* = 0.52; *I*^2^ = 0%), indicating that the effect of NHFT on invasive ventilation requirements remained consistent regardless of baseline hypoxemia severity (Fig. 16).

#### Need for non-invasive ventilation

**Figure 17.**
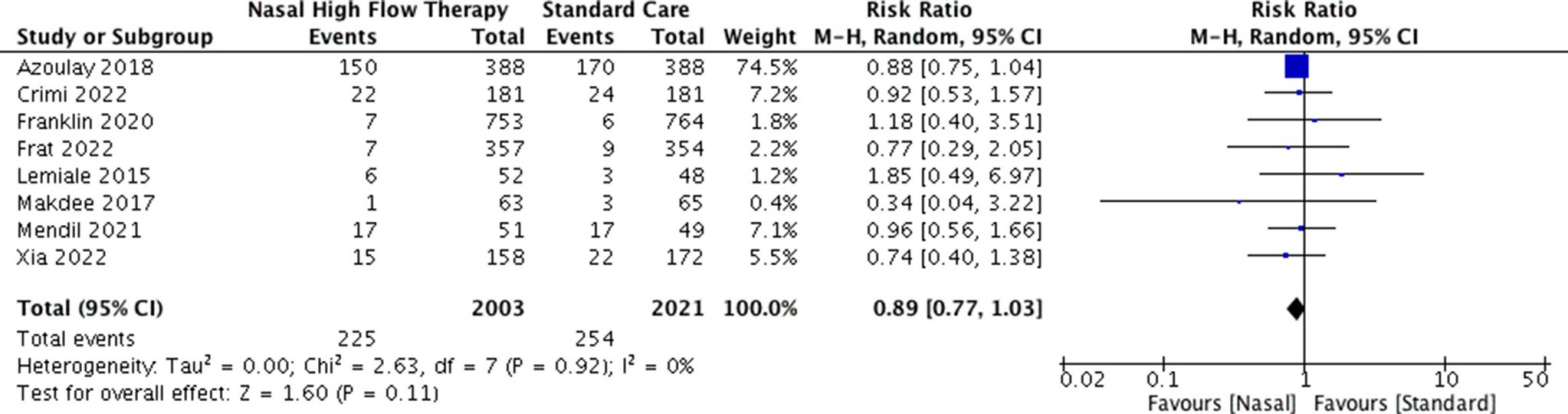
Forest plot showing the efficacy of nasal high-flow therapy versus standard oxygen therapy in hypoxemic respiratory failure in terms of need for non-invasive ventilation (RR: 0.89; 95% CI: 0.77–1.03; *P* = 0.11).

The need for NIV did not differ significantly between the NHFT and SOT groups (RR: 0.89; 95% CI: 0.77–1.03; *P* = 0.11). Heterogeneity between the studies was low (*I*^2^ = 0%) (Fig. 17).

#### Subgroup analysis

A subgroup analysis was performed to assess the effect of NHFT compared with SOT on the need for NIV, stratified by the severity of hypoxemia.

In patients with moderate hypoxemia, the pooled RR was 0.92 (95% CI: 0.68–1.24; *I*^2^ = 0%), indicating no significant difference between NHFT and SOT.

Among patients with severe hypoxemia, the pooled RR was 0.88 (95% CI: 0.74–1.04; *I*^2^ = 0%), similarly showing no significant difference.

**Figure 18.**
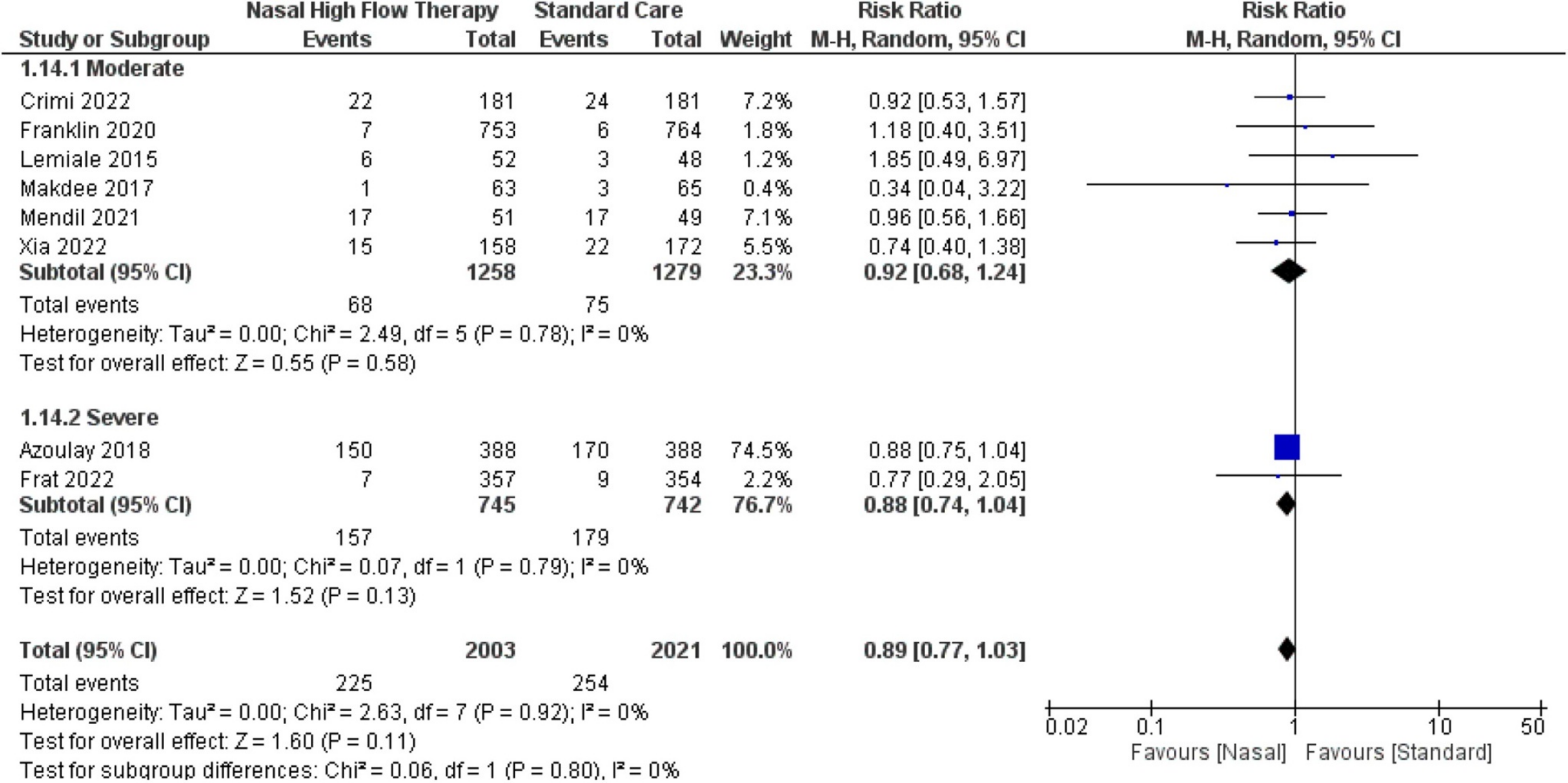
Subgroup analysis of need for noninvasive ventilation.

There was no significant subgroup difference based on hypoxemia severity (*P* = 0.80; *I*^2^ = 0%) (Fig. 18).

#### Dyspnea VAS score

**Figure 19.**
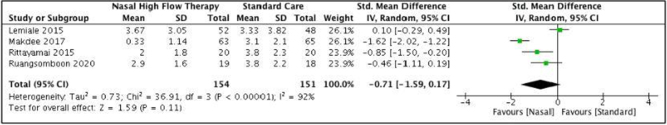
Forest plot showing the efficacy of nasal high-flow therapy versus standard oxygen therapy in hypoxemic respiratory failure in terms of dyspnea VAS scores (SMD: −0.71; 95% CI: −1.59 to 0.17; *P* = 0.11; *I*^2^ = 92%).

Dyspnea VAS scores did not differ significantly between the NHFT and SOT groups (SMD: –0.71; 95% CI: –1.59 to 0.17; *P* = 0.11; *I*^2^ = 92%) (Fig. 19). Sensitivity analysis excluding Lemiale *et al*^[^24^]^ and Makdee *et al*^[^35^]^ eliminated heterogeneity (*I*^2^ = 0%) and showed a significant difference favoring NHFT (SMD: –0.66; 95% CI: –1.12 to –0.20; *P* = 0.005) (Supplemental Digital Content Figure 5, available at: http://links.lww.com/MS9/B246)

#### Patient comfort (Comfort Scale)

**Figure 20.**
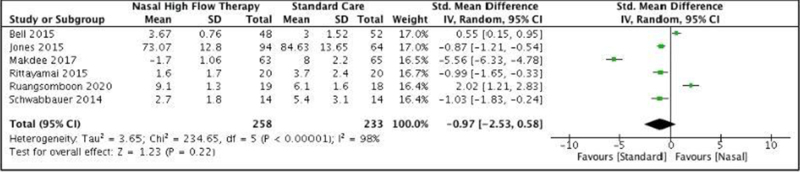
Forest plot showing the efficacy of nasal high-flow therapy versus standard oxygen therapy in hypoxemic respiratory failure in terms of Comfort Scale scores (SMD: −0.97; 95% CI: −2.53 to 0.58; *P* = 0.22; *I*^2^ = 98%).

Comfort Scale scores did not differ significantly between the NHFT and SOT groups (SMD: –0.97; 95% CI: –2.53 to 0.58; *P* = 0.22; *I*^2^ = 98%) (Fig. 20). Sensitivity analysis excluding Bell *et al*^[^28^]^, Makdee *et al*^[^35^]^, and Ruangsomboon *et al*^[^26^]^, removed heterogeneity (*I*^2^ = 0%) and showed a significant difference favoring NHFT (SMD: –0.91; 95% CI: –1.19 to –0.64; *P* < 0.00001) (Supplemental Digital Content Figure 4, available at: http://links.lww.com/MS9/B246)

#### Heart rate

**Figure 21.**
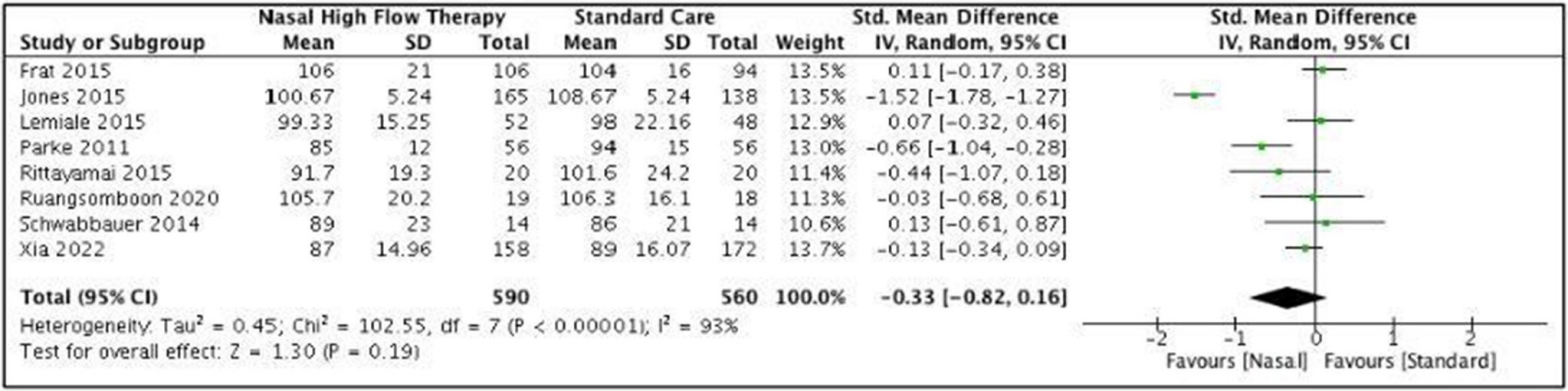
Forest plot showing the efficacy of nasal high-flow therapy versus standard oxygen therapy in hypoxemic respiratory failure in terms of heart rate (SMD: −0.33; 95% CI: −0.82 to 0.16; *P* = 0.19; *I*^2^ = 93%).

Heart rate did not differ significantly between the NHFT and SOT groups (SMD: –0.33; 95% CI: –0.82 to 0.16; *P* = 0.19; *I*^2^ = 93%) (Fig. 21). Sensitivity analysis excluding Jones *et al*^[^32^]^ removed heterogeneity (*I*^2^ = 0%) and still showed no significant difference (SMD: –0.04; 95% CI: –0.18 to 0.11; *P* = 0.59) (Supplemental Digital Content Figure 5, available at: http://links.lww.com/MS9/B246).

#### Subgroup analysis

Subgroup analysis of heart rate was performed based on the severity of hypoxemia.

Among patients with moderate hypoxemia, the pooled standardized mean difference (SMD) was −0.39 (95% CI: −0.94 to 0.16; *I*^2^ = 93%), while for those with severe hypoxemia, the SMD was 0.11 (95% CI: −0.17 to 0.38).

**Figure 22.**
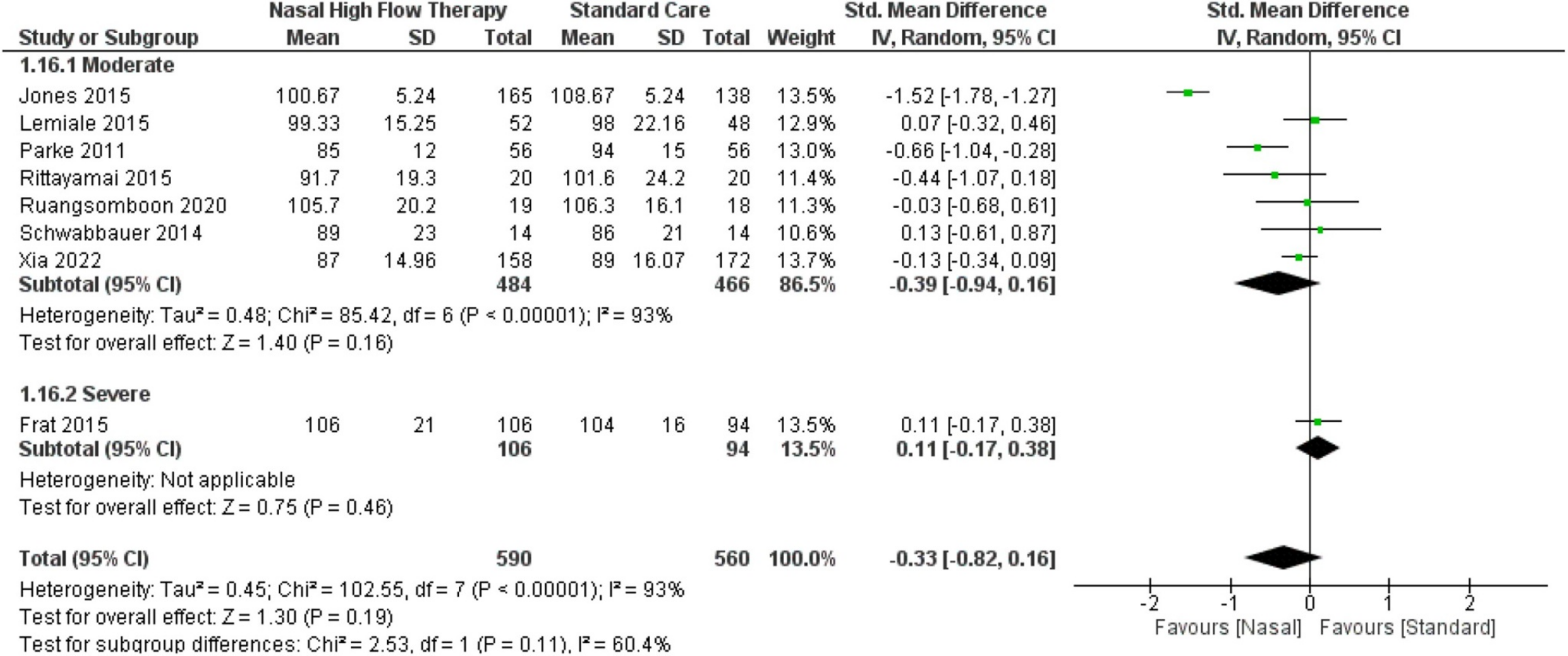
Subgroup analysis of heart rate.

There was no significant subgroup interaction (*P* = 0.11; *I*^2^ = 60.4%), indicating that heart rate outcomes were consistent across different severities of hypoxemia (Fig. 22).

#### Oxygen saturation (SpO2)

**Figure 23.**
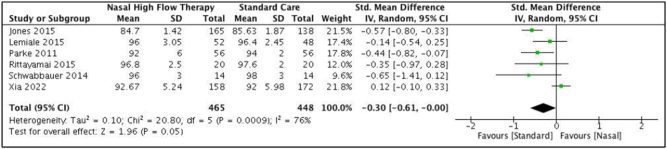
Forest plot showing the efficacy of nasal high-flow therapy versus standard oxygen therapy in hypoxemic respiratory failure in terms of oxygen saturation (SMD: −0.30; 95% CI: −0.61 to −0.00; *P* = 0.05; *I*^2^ = 76%).

Oxygen saturation (SpO₂) was significantly higher in the NHFT group compared to the SOT group (SMD: −0.30; 95% CI: −0.61 to −0.00; *P* = 0.05; *I*^2^ = 76%) (Fig. 23). Sensitivity analysis excluding Xia *et al*^[^25^]^ removed heterogeneity (*I*^2^ = 0%) and confirmed a significant difference favoring NHFT (SMD: −0.46; 95% CI: −0.62 to −0.29; *P* < 0.00001) (Supplemental Digital Content Figure 7, available at: http://links.lww.com/MS9/B246)

## Discussion

This systematic review and meta-analysis of 16 RCTs demonstrate that NHFT and SOT are comparable in efficacy and safety for managing acute hypoxemic respiratory failure. Our pooled analysis revealed no significant differences between the two interventions in primary clinical outcomes, such as length of hospital stay, 28-day and 90-day mortality, and in-hospital mortality. Secondary outcomes, including the incidence of cardiac arrest, need for intubation, length of ICU stay, need for invasive or NIV, dyspnea VAS score, patient comfort, and heart rate, were also statistically insignificant. The most consistent benefit of NHFT was a statistically significant improvement in oxygen saturation (SpO2), a finding that aligns with its known physiological advantages. These findings indicate comparable safety and efficacy profiles between NHFT and SOT, favoring NHFT as an acceptable alternative to conventional oxygen therapy across various clinical conditions.

The history of respiratory care has been phenomenal over the centuries. Initial attempts, such as manual bellows and negative-pressure ventilators (the iron lung), provided rudimentary assistance during epidemics such as poliomyelitis in the first half of the 20th century^[^39^]^. Following the identification of oxygen in the late 18th century and its therapeutic use in the early 1900s, SOT became the norm in hospital practice, usually delivered by nasal cannula or mask at low flow rates (≤6 L/min), though it was frequently insufficient to meet augmented inspiratory demand and often dried out the mucosa^[^40^]^. With advances in technology, NHFT was developed, providing heated, humidified oxygen at high flow rates (up to ≈60 L/min) with adjustable FiO₂, enabling dead-space washout, producing modest positive airway pressure, improving mucociliary clearance, and enhancing patient comfort^[^11,34^]^. Over the decades, sufficient clinical evidence has been gathered in various hospital settings and patient populations. Meta-analyses have confirmed that NHFT decreases intubation rates compared with SOT in acute hypoxemic respiratory failure, without significantly altering mortality^[^17,41^]^. In addition, NHFT delivered after planned extubation is associated with significantly lower reintubation rates and reduced post-extubation respiratory failure compared to SOT^[^42,43^]^. Although SOT and NHFT both attempt to maximize oxygenation, NHFT’s simultaneous physiological effects – augmented gas exchange, mild PEEP effect, and improved patient tolerance–render it a valuable adjunct, particularly in moderate to severe hypoxemia when conventional oxygen therapy may not be sufficient.

In evaluating the effect of NHFT compared to SOT on the length of hospital stay in patients with acute hypoxemic respiratory failure, our findings align with several high-quality studies and meta-analyses that reported no significant difference between these two modalities, notably Rochwerg *et al*^[^17^]^. Other recent systematic reviews have consistently shown that, in this patient group, NHFT does not confer a clear advantage over SOT in reducing hospital stay duration. These results suggest that, while NHFT is a safe and well-tolerated alternative, its impact on hospitalization length may be comparable to that of SOT in acute hypoxemic respiratory failure. Expanding the discussion to other clinical scenarios, the literature reveals a more favorable role for NHFT in specific populations. For instance, in patients recovering from cardiac surgery or in postoperative settings, Lu *et al*^[^44^]^ and Zochios *et al*^[^45^]^ reported that NHFT can lead to a shorter hospital stay than SOT, likely due to improved respiratory support and patient comfort. However, these benefits appear to be context-dependent and are not universally observed in all patient groups.

Similarly, our results regarding 28-day mortality were consistent with those of Rochwerg *et al*’s meta-analysis, which found no mortality benefit of NHFT compared with SOT^[^17^]^. Wang *et al* also observed comparable mortality rates in COVID-19 patients treated with NHFT and SOT, supporting the absence of a survival advantage^[^46^]^. While some observational studies, such as that by Song *et al*, suggested possible mortality benefits in specific subgroups, such as sepsis and ARDS, these findings were not consistently replicated in larger populations^[^47^]^. Seow *et al* further concluded that NHFT does not substantially affect 28-day mortality compared to SOT^[^48^]^.

Regarding 90-day mortality, Marjanovic *et al* similarly demonstrated no significant difference between NHFT and SOT, with pooled data from multiple trials showing comparable outcomes^[^49^]^. This aligns with the Cochrane meta-analysis by Lewis *et al*, which found that NHFT probably makes little or no difference in mortality relative to SOT^[^50^]^. The lack of mortality benefit may reflect the fact that, although NHFT improves oxygenation and comfort, it does not alter the progression of underlying fatal conditions, such as multi-organ failure or severe infection, as highlighted by Raoof *et al*^[^51^]^.

Furthermore, our evidence of cardiac arrest is in agreement with the results of a previous study by Hernández *et al*, who found no significant difference in severe adverse events, including cardiac arrest, between NHFT and SOT recipients during planned extubation in intensive care units^[^52^]^. Similarly, the FLORALI trial by Frat *et al* found comparable rates of cardiac arrest in patients with acute hypoxemic respiratory failure treated with NHFT and SOT^[^30^]^. These findings are supported by the meta-analytic evidence of Rochwerg *et al*, which indicates that patients receiving NHFT are not at greater risk of severe events, such as cardiac arrest, than those receiving SOT^[^17^]^.

Consistent with our findings on ICU length of stay, Liesching and Lei reported no difference in ICU length of stay between NHFT- and SOT-receiving ICU patients in their meta-analysis of ICU patients, indicating comparability between the two in this regard^[^53^]^. Similarly, in a systematic review of adult patients with COVID-19, Arruda *et al* noted no statistically significant variation in ICU stay between NHFT and conventional oxygen therapy, again pointing to the applicability of these findings to other patient groups and disease conditions^[^54^]^.

In addition to our evidence on intubation, Ni *et al* conducted a meta-analysis that found no difference in intubation rates between NHFT and SOT in patients with acute respiratory failure^[^41^]^. A RCT by Pr *et al* also concluded that NHFT did not substantially reduce the rate of mechanical ventilation compared with noninvasive ventilation (NIV) in patients with severe COVID-19^[^55^]^. These studies show that while NHFT may have certain physiological benefits, its efficacy in reducing intubation rates remains uncertain across different patient categories.

In contrast to other outcomes, our results demonstrated a statistically significant improvement in oxygen saturation (SpO₂) with NHFT compared to SOT in patients with acute hypoxemic respiratory failure, a finding that resonates with the randomized trial of Emami-Ardestani *et al*^[^56^]^ which observed a higher median SpO₂ in the NHFT group, alongside a notable reduction in intubation risk. Similarly, Hernández *et al*^[^52^]^ reported an absolute increase in SpO₂ with NHFT in low-risk patients following planned extubation in the ICU, highlighting the clinically relevant gains for patients with marginal saturations. Meta-analyses focused on high-risk procedures, such as bronchoscopy and gastrointestinal endoscopy, have also consistently shown that NHFT raises SpO₂ and significantly reduces the incidence of hypoxemia^[^57,58^]^. These improvements are attributed to NHFT's ability to deliver high and stable flows, generate low-level positive airway pressure, and facilitate dead-space washout, thereby optimizing oxygenation. Our findings align with the growing body of evidence supporting NHFT’s superiority in elevating oxygen saturation during the acute phase of hypoxemic respiratory failure.

NHFT therapy delivers a tempered, humidified air-oxygen mixture at flow rates above a patient’s inspiratory demand, maintaining a constant and predictable fraction of inspired oxygen (FiO₂) despite respiratory variability^[^40^]^. By flushing the nasopharyngeal space, NHFT minimizes the entrainment of ambient air and reduces CO_2_ rebreathing^[^59^]^. Body temperature and total humidity gas delivery maintain mucosal function, gas conditioning, and minimize insensible water loss, enhancing patient comfort and airway integrity[^[^40^]^. Notably, NHFT has several pathophysiological benefits that enhance respiratory function. High flow promotes anatomical dead space clearance, maximizes alveolar ventilation, and minimizes the effective dead space-to-tidal volume ratio[^[^59^]^. It also creates a mild, flow-dependent positive airway pressure that is beneficial for alveolar recruitment and stabilization^[^13^]^. Furthermore, conditioned gas enhances mucociliary clearance and decreases airway resistance. These combined effects minimize the work of breathing, improve oxygenation, and enhance patient tolerance in various forms of respiratory failure^[^13,40^]^.

The stated review uses sophisticated synthesis practices to represent the rapid development of Artificial Intelligence (AI) in the medical domain. It is important to note that Large Language Models are transforming research and clinical decision-making processes, as demonstrated by a worldwide study of their effects^[^60^]^. Our results on the equivalence of NHFT/SOT can serve as a valuable source of information for the future implementation of AI. The methodological framework developed here should help healthcare professionals and clinicians trust our method regarding the reliability of the approach, which will facilitate the creation of specific and personalized respiratory support methods in critical care. This interlocking forms an essential methodological base for wise and personalized suggestions in critical care setups.

### Limitations and strengths

This meta-analysis included sixteen high-quality RCTs, offering a comprehensive evaluation of NHFT versus SOT across multiple clinically relevant outcomes, including mortality, length of stay, intubation rates, and oxygen saturation. The diversity of patient populations and clinical settings, from emergency departments to intensive care units, enhances the generalizability of the findings, whereas the consistency across primary and secondary endpoints strengthens confidence in the comparable safety and efficacy profiles of both therapies. However, limitations arise from the heterogeneity in study designs, patient groups, and outcome definitions, which may mask subtle benefits in specific subpopulations. Variability in intervention protocols and potential publication bias could have influenced the pooled results. Although most outcomes showed no significant difference, the possibility of underpowered analyses for rare events, such as cardiac arrest, cannot be excluded. Furthermore, evolving respiratory care practices and emerging diseases may limit the applicability of these findings to future clinical settings. Overall, while NHFT appears to be a safe and effective alternative to SOT, individualized clinical judgment remains essential.

## Conclusion

In conclusion, this comprehensive meta-analysis shows that NHFT and SOT have comparable safety and efficacy profiles for managing acute hypoxemic respiratory failure, with no significant differences in mortality, length of hospital or ICU stay, or need for invasive ventilation. Although NHFT does not confer a mortality advantage over SOT, it remains a safe and well-tolerated modality that demonstrated a statistically significant increase in oxygen saturation compared to SOT; however, this absolute difference was minimal and not clinically meaningful. This finding should be interpreted as reflecting improved consistency in oxygen delivery rather than a clinically significant physiological advantage. NHFT provides better oxygenation stability and patient comfort. These qualities may justify its use as a preferred supportive strategy in selected patients with acute hypoxemic respiratory failure. While these findings support NHFT, the observed heterogeneity and evolving respiratory care practices suggest that further research is required. Future studies should focus on identifying specific patient subgroups who may derive the greatest benefit from NHFT, optimizing flow settings and timing, and evaluating long-term outcomes in the context of emerging respiratory diseases. Such targeted investigations will help refine clinical guidelines and enhance personalized respiratory-support strategies.

## Data Availability

All data used in this systematic review and meta-analysis are from publicly available published sources.

## Research registration unique identifying number (UIN)

Prospero Registration link: http://crd.york.ac.uk/PROSPERO/view/CRD420251103622

Registration Number: .CRD420251103622.
